# Diagnostic accuracy of circulating exosomal circRNAs in malignances: A meta-analysis and systematic review

**DOI:** 10.1097/MD.0000000000033872

**Published:** 2023-05-26

**Authors:** Xia Yuan, Ye Mao, Shuangyan Ou

**Affiliations:** a Gastroenterology and Urology Department Ⅱ, Hunan Cancer Hospital/the Affiliated Cancer Hospital of Xiangya School of Medicine, Central South University, Changsha, P.R. China; b Clinical Research Center For Gastrointestinal Cancer In Hunan Province, Changsha, P.R. China; c Department of Oncology, The Second Affiliated Hospital of Nanchang University, Nanchang, Jiangxi, P.R. China.

**Keywords:** cancer, circular RNAs, diagnosis, exosome, meta-analysis

## Abstract

Pathogenesis of malignant tumors are often accompanied by aberrant expression of circular RNAs (circRNAs), indicating the potential diagnostic value of circRNAs in tumors. CircRNAs have been found to be enriched, stable and ubiquitous in serum and plasma exosomes. The study aims at evaluating the diagnostic performance of circulating (plasma and serum) exosomal circRNA in different types of cancer by synthesis of published data.

**Methods:**

A comprehensive literature search was conducted in PubMed, Embase, Medline and the Web of Science databases to identify potentially eligible studies published before April 2021. We conducted the meta-analysis following the Preferred Reporting Items for Systematic Reviews and Meta-Analyses recommendations.

**Results:**

Eleven articles comprising 21 studies were included, and a total of 1609 cases and 1498 controls were evaluated. Six types of cancer were involved in these studies, including lung cancer, hepatocellular carcinoma, colorectal cancer, gastric cancer, multiple myeloma and osteosarcoma. The pooled sensitivity and specificity were 0.72 (95% confidence interval [CI], 0.62–0.81) and 0.83 (95% CI, 0.78–0.88), respectively. Summary receiver operating characteristic curve was constructed and the pooled value of area under curve was 0.86 (95% CI, 0.83–0.89), indicating a favorable diagnostic efficacy of circulating exosomal circRNAs in malignancies.

**Conclusions:**

In conclusion, our study evaluated the diagnostic power of circulating exosomal circRNAs in 6 types of cancer by synthesis of published data comprising 21 studies from eleven articles. The pooled analysis provided the evidence supporting circulating exosomal circRNAs as a promising noninvasive diagnostic biomarkers for malignancies.

## 1. Introduction

Cancer ranks among the leading causes of death around the world^[1]^ and the therapy approaches have been explored extensively from chemotherapy, surgery, radiotherapy, interventional therapy, targeted therapy to immunotherapy.^[2]^ Nevertheless, the clinical effect of anti-tumor therapies on the improvement in survival is far from satisfaction, which was most commonly attributed to the advanced stage at diagnosis.^[3]^ Early-stage cancer does not produce obvious symptoms and therefore new diagnostic techniques are indispensable for early diagnosis, ultimately improving the life quality and survival rate of cancer patients. Over the last decade, the liquid biopsy technique attracted increasing attention and many researches were focused on the identification and validation of circulating biomarkers for the early diagnosis of tumors. The advantages of liquid biopsy over traditional histological biopsy include non-invasiveness, simplicity, speed and dynamic monitoring.^[4]^ At present, however, diagnostic efficiency of the established biomarkers such as CEA and CA199 remains lower than desired, and it is urgent to explore novel stable and reliable cancer biomarkers.^[5]^

In the recent years, exosome and its diverse internal cargos represent an emerging field in cancer research. Exosomes are lipid bilayer-enclosed extracellular vesicles with a size range of 40 to 160 nm in diameter (averaging 100 nm) and they are secreted by almost all cell types.^[6,7]^ Exosomes are transferred from donor to recipient cells, revealing a novel mechanism of intercellular communication,^[8]^ in which way exosomes participate in a wide range of biological processes and contribute to cancer initiation and progression.^[9]^ The function of exosome as “communication shuttles” is ascribed to its diverse internal cargos of bioactivity, including lipids, proteins and nucleic acids.^[10,11]^ Non-coding RNAs including miRNAs, long non-coding RNAs, and circular RNAs (circRNAs) encapsulated in exosomes can be delivered to the recipient cells,^[12]^ involving in oncogenic cell transformation, cell apoptosis and proliferation, angiogenesis, metastasis, drug resistance and antitumor immunity.^[13,14]^

Distinguished from the traditional linear RNA, circRNA is a novel non-coding RNA molecule with a covalently closed loop.^[15]^ Since the lack of 5’-end cap structure and 3’ poly-A tail structure provides resistance to RNA exonuclease, circRNAs have greater stability than linear RNA with a half-life up to 48 hours.^[16]^ CircRNAs primarily function as post-transcriptional regulators through regulating downstream gene expression by sponging miRNAs at miRNA binding sites and through interacting with RNA-associated proteins.^[17]^ Pathogenesis and progression of tumors are often accompanied by aberrant expression of circRNAs in plasma and tumor tissues, indicating the potential diagnostic value of circRNAs in tumors.^[18]^

With the rapid development of bioinformatics and widespread use of high-throughput sequencing technology, circRNAs have been found to be enriched, stable and ubiquitous in exosomes.^[12]^ CircRNAs are selectively sorted into exosomes via complex passive and active mechanisms. Previous studies have reported that circRNAs are enriched in exosomes, even when they are down-regulated in the parental cells.^[8,12,19,20]^ Thus, the diagnostic value of plasma or serum exsomal circRNAs may be further enhanced.^[21]^ Recently, a small number of studies have assessed the diagnostic performance of plasma or serum exosomal circRNA in malignancies, reporting different diagnostic accuracies.^[22,23]^ Our study therefore aims at evaluating the diagnostic performance of plasma or serum exosomal circRNA for malignancies by synthesis of published data.

## 2. Methods

### 2.1. Search strategy

We conducted the meta-analysis following the Preferred Reporting Items for Systematic Reviews and Meta-Analyses recommendations (http://prismastatement.org) without a specific protocol for the systematic review. A comprehensive literature search was conducted in PubMed, Embase, Medline and the Web of Science databases to identify potentially eligible studies published before April 2021. The literature search strategy and the criteria for study eligibility followed the framework of Population-Intervention-Comparator-Outcomes. The population was defined as patients with cancer. The intervention was defined as differential expression levels of plasma or serum exosomal circRNAs and the comparator as the gold standard was pathological diagnosis of biopsy or surgical specimen. The outcome included receiver operating characteristic (ROC) curves, sensitivity (Sen), specificity (Spe), area under curve (AUC), negative diagnostic likelihood ratio (DLR−) and positive diagnostic likelihood ratio (DLR+) with corresponding 95% confidence intervals (CI). We used the following search terms: “circular RNAs OR circRNAs OR hsa_circ OR noncoding RNA” AND “cancer OR carcinoma OR tumor OR neoplasm OR malignancy OR malignant neoplasm” AND “diagnosis OR biomarker OR prediction OR sensitivity OR specificity OR ROC curve OR AUC” AND “exosomes OR exosome OR extracellular vesicles.” In addition, the reference lists were manually searched to identify additional eligible studies. Since the study was based on synthetic analysis of previously published data, no ethical approval or patient consent was required.

### 2.2. Eligibility criteria and study selection

Enrolled studies in the meta-analysis satisfied the following criteria: study cases were patients diagnosed with all types of malignancies by histopathology of biopsy or surgery specimens; studies included healthy or noncancerous population as controls; serum or plasma exosomes were isolated and detected for circRNA expression; Exosomal circRNAs in cases and controls were differentially expressed; studies provided enough information to estimate true-positive (TP), false-positive (FP), false-negative (FN), and true-negative (TN) values. Studies were excluded from the meta-analysis if meeting the following criteria: studies were not published in English; study reported duplicate or overlapping data; sample size in the study was fewer than 20 patients. Two investigators, X.Y. and Y.M., screened relevant abstracts and reviewed eligible full-texts independently, and then made the decision to include or exclude article. In the case of discordance, the third author, X.Y. was responsible for coordinating disagreements.

### 2.3. Data extraction

We collected the following information from the selected literature: author name, publication year, country, cancer type, circRNA, number of cases and controls, exosome isolation method and exosome identification methods. Then, diagnostic accuracy variables such as sensitivity, specificity and AUC values were collected. For studies that had not shown the corresponding results, we used a digitizing software-Engauge Digitizer to read ROC curves and extract matching sensitivity and specificity values from the ROC curves. Engauge Digitizer was extensively used in previous studies to get data in the curves^[24]^ and in our previous study, we used Engauge Digitizer to read the Kaplan–Meier curves to obtain information of survival.^[25]^ In addition, a method named Youden’s index has been developed to determine the optimal sensitivity and specificity values for quantitative diagnostic tests. Youden’s index is defined as *sensitivity + specificity−1*^[26]^ and the maximum of Youden’s index (J_max_) corresponds to the point on the ROC curve with optimal sensitivity and specificity. Based on the J_max,_ we obtained the values of Spe and Sen that were not accessible in the articles from the ROC curves using Engauge Digitizer.

For each study, 2 × 2 contingency tables were obtained with values of TN, FN, TP and FP. For studies that had not provided theses data, we calculated the corresponding values based on the sensitivity, specificity, numbers of cases and controls. DLR+ was calculated as Sen/(1−Spe), DLR− was calculated as (1−Sen)/Spe, and diagnostic odds ratios (DOR) is calculated as DLR+/DLR−. The 2 investigators, X.Y. and Y.M, conducted data extraction independently, and the disagreements were settled by a face-to-face consultation with the third author Shuangyan Ou.

### 2.4. Quality assessment

The quality of individual study included in the meta-analysis was assessed according to the Quality Assessment of Diagnosis Accuracy Studies (QUADAS-2) criteria (http://prisma-statement.org).^[27]^ The QUADAS-2 evaluated risk of bias and clinical applicability concerns following 14 items by answering *yes, no* and *unclear,* which scores −1, 1 and 0, respectively. The items involve 4 aspects: patient selection, index test, reference standard, and flow and timing. All components will be evaluated in terms of bias risk, and the first 3 components will be evaluated in terms of clinical applicability. The 2 investigators, X.Y. and Y.M., assessed the quality independently, and RevMan 5.3 software was used to present the assessment results.

### 2.5. Statistical analysis

The diagnostic performance of plasma exosomal circRNAs for malignancies was analyzed by Stata software version 11.1 (STATA Corporation, College Station, TX). The Q statistic of the chi-squared value test and the inconsistency index (*I*^2^) were used to estimate the heterogeneity among the enrolled studies.^[28]^ *P* value ≤ .1 or *I*^2^ ≥ 50% indicates the existence of significant heterogeneity, and in the case, a random effects model was adopted.^[29]^ If *P* > 0.1 and *I*^2^ < 50%, it was considered that the heterogeneity of included studies was lower, and the fixed effects model was adopted for the pooled analysis.^[30]^ Moreover, the bivariate boxplot played an auxiliary role in heterogeneity evaluation.

The bivariate random-effects regression model was adopted to pool the diagnostic effect values based on 2 × 2 contingency tables with TP, TN, FP and FN. Pooled analysis included Sen, Spe, DLR+, DLR− and DOR with 95% CI.^[31]^ The summary receiver operating characteristic (SROC) curve was plotted to calculate the pooled AUC value.^[32]^ Moreover, meta-regression analysis and subgroup analysis were performed to identify the source of heterogeneity.

The publication bias was assessed using Deek’s funnel plot asymmetry test, and the unequally distributed visual funnel plot or *P* value ≤ 0.5 indicates the existence of significant publication bias in the study.^[33]^ To further explore the clinical significance of circulating exosomal circRNAs in cancer diagnosis, we plotted the Fagan plot to explain the interaction between pretest probability, likelihood ratio, and post-test probability. Also, a likelihood ratio scattergram was performed to estimate the clinical applicability of circulating exosomal circRNAs in cancer diagnosis.^[14]^

## 3. Results

### 3.1. Literature search and selection of studies

**Figure 1.**
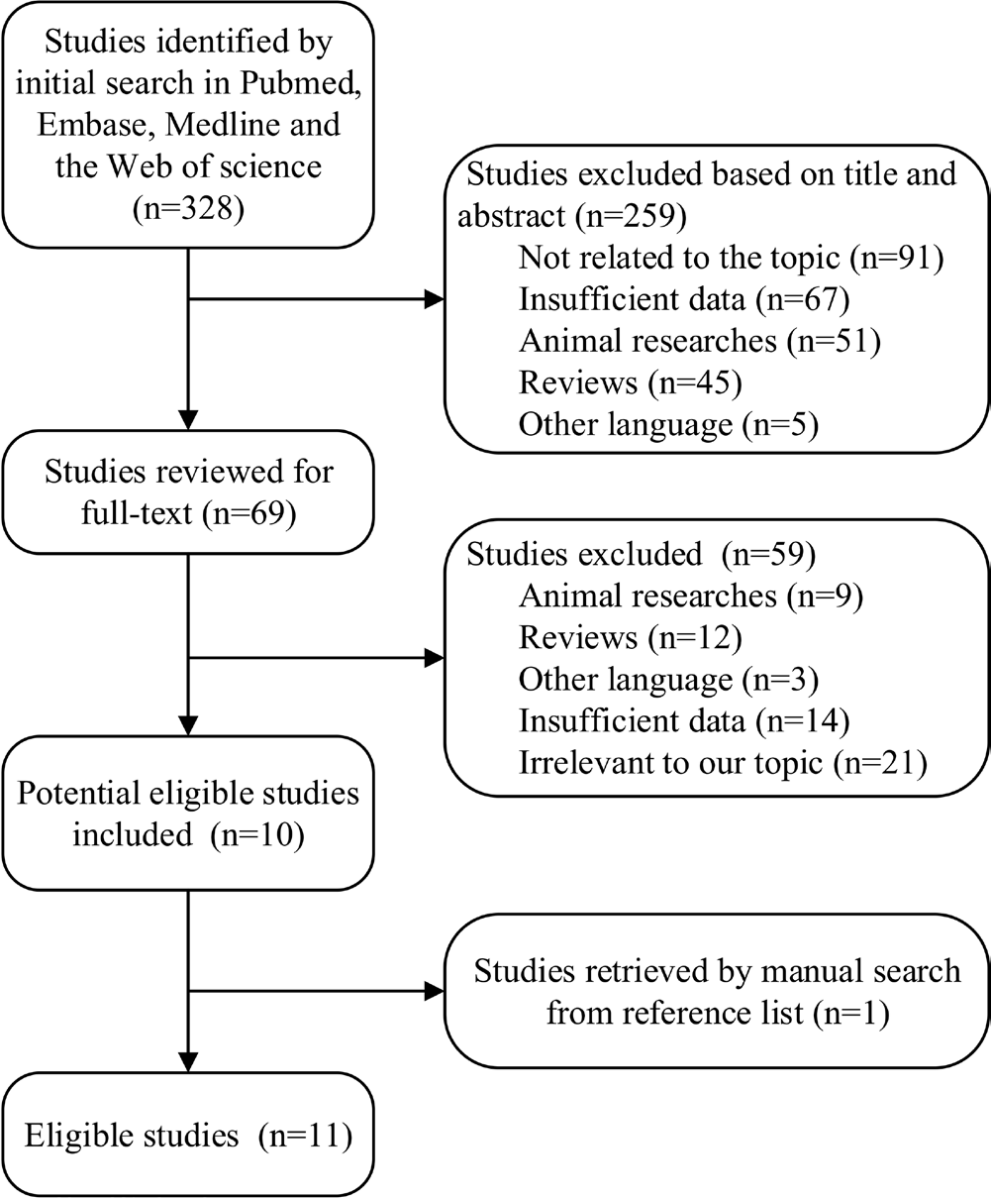
Flow chart of selection process for eligible studies.

The procedure for selecting eligible articles in this study is shown in Figure 1. A comprehensive literature search in PubMed, Embase, Medline and the Web of Science databases identified 328 articles after excluding repetition. A number of 259 articles were excluded after screening abstracts one by one. A total of 69 full-texts were reviewed, of which 10 articles were finally included. One article was identified by manually checking the reference lists, and therefore 11 eligible articles^[18,22,23,34–41]^ were enrolled in the meta-analysis.

### 3.2. Study characteristics

The main characteristics of all included articles were listed in Table 1. Eleven articles comprising 21 studies were included which were performed in China between 2018 and 2021. Plasma samples were used in 7 studies from 4 articles and 14 studies from 7 articles used serum as samples. The sample size among these studies ranged from 55 to 285, and sample sizes of 5 studies were less than 100, 16 more than 100. Among the 11 articles, a total of 1609 cases and 1498 controls were evaluated. Fourteen kinds of exosomal circRNAs were detected and evaluated, including circ_0056285, circSATB2, circ_0014235, circ_0025580, circMYC, circ_0051443, circ_0047921, circ_0056285, circ_0007761, circMEMO1, circ_0065149, circ-0004771, circ_0101802 and circ_0070396. Four articles^[18,22,34,37]^ investigated the molecular mechanism of circRNA expression pattern and circRNA-miRNA-mRNA network in the pathogenesis of cancer. Six types of cancer were involved in these studies, including lung cancer (11 studies from 4 articles), hepatocellular carcinoma (4 studies from 2 articles), colorectal cancer (3 studies from 2 articles), gastric cancer (1 study from 1 article), multiple myeloma (1 study from 1 article), and osteosarcoma (1 study from 1 article). The vast majority of these studies evaluated the diagnostic performance of exosomal circRNAs for cancers without stratification by tumor clinical stage, and only 2 studies were conducted in patients with early-stage gastric cancer^[38]^ and colorectal cancer,^[39]^ respectively.

**Table 1:** The main characteristics of included studies.

Author	Year	Region	circRNA	Samples	Cancer type	Case types	Control types	Case number	Control number	QUADAS score
Huo et al	2021	China	circ_0056285	Serum	Osteosarcoma	Patients	Healthy volunteers	35	35	3
Zhang et al	2020	China	circSATB2	Serum	Non-small cell lung cancer	Patients	Healthy volunteers	83	95	4
Wang et al	2020	China	circ_0014235	Plasma	Lung squamous cell carcinoma	Patients	Healthy volunteers	30	30	3
Wang et al	2020	China	circ_0025580	Plasma	Lung squamous cell carcinoma	Patients	Healthy volunteers	30	30	3
Luo et al	2020	China	circMYC	Serum	Multiple myeloma	Patients	Healthy volunteers	122	54	4
Chen et al	2020	China	circ_0051443	Plasma	Hepatocellular carcinoma	Patients	Healthy volunteers	60	60	4
Xian et al	2019	China	circ_0047921	Serum	Non-small-cell lung cancer	Patients	Healthy volunteers	120	165	6
Xian et al	2019	China	circ_0056285	Serum	Non-small-cell lung cancer	Patients	Healthy volunteers	120	165	6
Xian et al	2019	China	circ_0007761	Serum	Non-small-cell lung cancer	Patients	Healthy volunteers	120	165	6
Xian et al	2019	China	circ_0047921	Serum	Non-small-cell lung cancer	Patients	Healthy volunteers	62	95	6
Xian et al	2019	China	circ_0056285	Serum	Non-small-cell lung cancer	Patients	Healthy volunteers	62	95	6
Xian et al	2019	China	circ_0007761	Serum	Non-small-cell lung cancer	Patients	Healthy volunteers	62	95	6
Xian et al	2019	China	circ_0047921	Serum	Non-small-cell lung cancer	Patients with non-small-cell lung cancer	Patients with COPD	63	58	6
Ding et al	2020	China	circMEMO1	Serum	Non-small cell lung cancer	Patients	Healthy volunteers	30	25	3
Shao et al	2019	China	circ_0065149	Plasma	Gastric cancer	Patients with early gastric cancer	Healthy volunteers	39	41	3
Pan et al	2019	China	circ-0004771	Serum	Colorectal cancer	Patients with colorectal cancer	Healthy volunteers	110	35	6
Pan et al	2019	China	circ-0004771	Serum	Colorectal cancer	Patients with stage I/II colorectal cancer	Healthy volunteers	70	35	6
Xie et al	2020	China	circ_0101802	Serum	Colorectal cancer	Patients	Healthy volunteers	58	58	5
Lyu et al	2021	China	circ_0070396	Plasma	Hepatocellular carcinoma	Patients with hepatocellular carcinoma	Healthy volunteers	111	54	5
Lyu et al	2021	China	circ_0070396	Plasma	Hepatocellular carcinoma	Patients with hepatocellular carcinoma	Patients with chronic hepatitis B	111	50	5
Lyu et al	2021	China	circ_0070396	Plasma	Hepatocellular carcinoma	Patients with hepatocellular carcinoma	Patients with HBV related liver cirrhosis	111	58	5

NA = not acquired, qRT-PCR = quantitative real-time polymerase chain reaction.

The values regarding diagnostic effect were presented in Table 2, including TP, TN, FP and FN, Sen, Spe, AUC, DOR. Five articles provided information of Sen and Spe values,^[37–41]^ while the Sen and Spe values were obtained by reading ROC curves through digitizing software-Engauge Digitizer based on the J_max_ for the other 6 articles.^[18,22,23,34–36]^ The information of exosomes in enrolled studies was collected in Table 3, which included exosome isolation method, exosomal content, exosome transmission electron microscope (TEM), exosome nanoparticle tracking analysis, exosome proteins and exosome diameter range. In these studies, plasma or serum exosomes were extracted by exosome isolation kit or ultracentrifugation, of which 7 articles^[18,22,23,34,39–41]^ performed scientific exosomal identification, including TEM, nanoparticle tracking analysis and Western blot assay of exosome marker.

**Table 2:** The values regarding diagnostic effect of included studies.

Author	Year	TP	FP	FN	TN	Sen	Spe	AUC	DOR	Cutoff
Huo et al	2021	22	7	13	28	0.628	0.800	0.778	6.746	NA
Zhang et al	2020	39	21	44	74	0.470	0.779	0.660	3.123	NA
Wang et al	2020	23	3	7	27	0.755	0.890	0.825	24.894	NA
Wang et al	2020	21	4	9	26	0.684	0.876	0.800	15.209	NA
Luo et al	2020	98	4	24	50	0.807	0.918	0.924	46.665	1.830
Chen et al	2020	40	6	20	54	0.670	0.900	0.809	18.272	NA
Xian et al	2019	112	66	8	99	0.931	0.600	0.757	20.376	NA
Xian et al	2019	47	22	73	143	0.391	0.868	0.625	4.207	NA
Xian et al	2019	63	17	57	148	0.526	0.899	0.750	9.878	NA
Xian et al	2019	60	48	2	47	0.966	0.494	0.745	27.617	NA
Xian et al	2019	41	12	21	83	0.663	0.875	0.849	13.704	NA
Xian et al	2019	25	6	37	89	0.404	0.937	0.661	10.178	NA
Xian et al	2019	62	15	1	43	0.985	0.742	0.890	193.476	NA
Ding et al	2020	17	1	13	24	0.567	0.960	0.760	31.389	NA
Shao et al	2019	19	4	20	37	0.487	0.902	0.640	8.738	6.430
Pan et al	2019	89	6	21	29	0.809	0.829	0.880	20.489	NA
Pan et al	2019	57	9	13	26	0.814	0.743	0.810	12.671	NA
Xie et al	2020	52	18	6	40	0.897	0.690	0.826	19.384	NA
Lyu et al	2021	69	1	42	53	0.622	0.982	0.857	87.152	NA
Lyu et al	2021	85	16	26	34	0.766	0.680	0.774	6.948	NA
Lyu et al	2021	52	11	59	47	0.469	0.810	0.661	3.765	NA

AUC = area under curve values, DOR = diagnostic odds ratios, FN = false-negative, FP = false-positive, Sen = sensitivity, Spe = specificity, TN = true-negative, TP = true-positive.

**Table 3:** The information of exosomes in enrolled studies.

Author	Year	Exosome isolation method	Exosomal content	TEM	NTA	Exosome proteins	Exosome diameter range
Huo et al	2021	Ultracentrifugation	circRNAs	Yes	Yes	CD61, CD81 and TSG101	75–150 nm
Zhang et al	2020	Total Exosome Isolation Reagent	circRNAs	Yes	Yes	TSG101, CD9 and CD63	≤200 nm
Wang et al	2020	Hieff exosome isolation kit	circRNAs	None	None	None	None
Luo et al	2020	Exosome isolation kit	circRNAs	Yes	None	None	None
Chen et al	2020	ExoQuick exosome precipitation solution	circRNAs	Yes	None	TSG101 and CD63	50–150 nm
Xian et al	2019	SBI ExoQuick exosome precipitation solution	circRNAs	Yes	Yes	CD81, CD63 and ALIX	30–150 nm
Ding et al	2020	ExoQuick precipitation kit	circRNAs	Electron microscopy	None	CD9, CD81 and TSG101	None
Shao et al	2019	Total exosome isolation reagent	circRNAs	None	None	None	None
Pan et al	2019	Invitrogen™ Total Exosome Isolation Kits	circRNAs	Yes	Yes	CD63 and TSG101	10–200 nm
Xie et al	2020	ExoQuick exosome precipitation solution	circRNAs	Yes	Yes	CD9 and TSG101	50–150 nm
Lyu et al	2021	exoEasy Maxi Kit	circRNAs	Yes	Nanoflow cytometer analysis	CD9, CD63 and TSG101	50–200 nm

NTA = nanoparticle tracking analysis, TEM = transmission electron microscope.

### 3.3. Assessment of study quality

**Figure 2.**
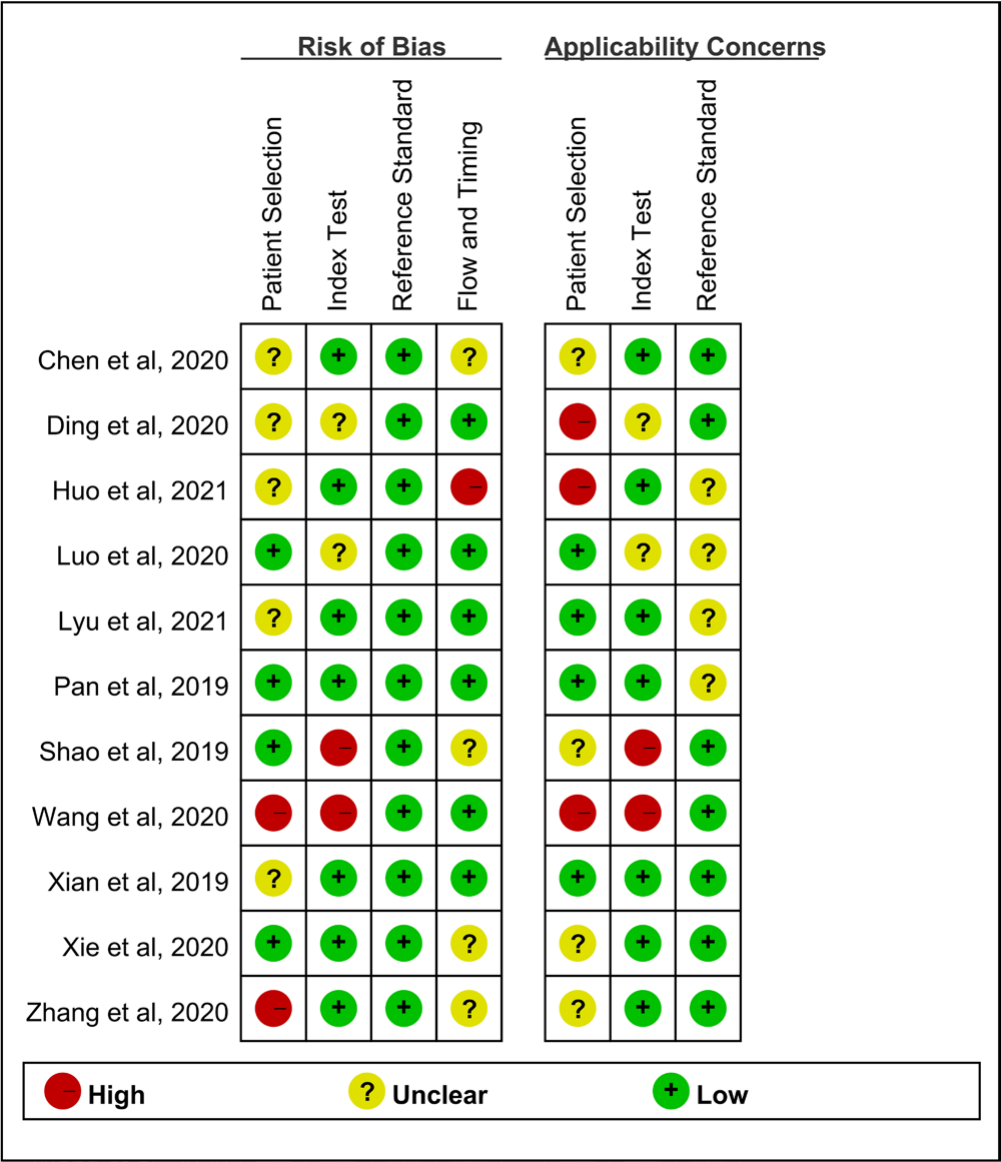
Overall quality assessment of all included articles using the QUADAS-2 tool.

QUADAS-2 quality assessment for the 11 included articles were shown in Figure 2, and the QUADAS scores were summarized in Table 1. We defined the study as a high-quality one if scoring greater than 4 and the study scoring lower than 4 was considered low-quality. The overall quality of included articles was favorable, of which 7 articles were high qualified and four were low-quality.

### 3.4. Heterogeneity test

**Figure 3.**
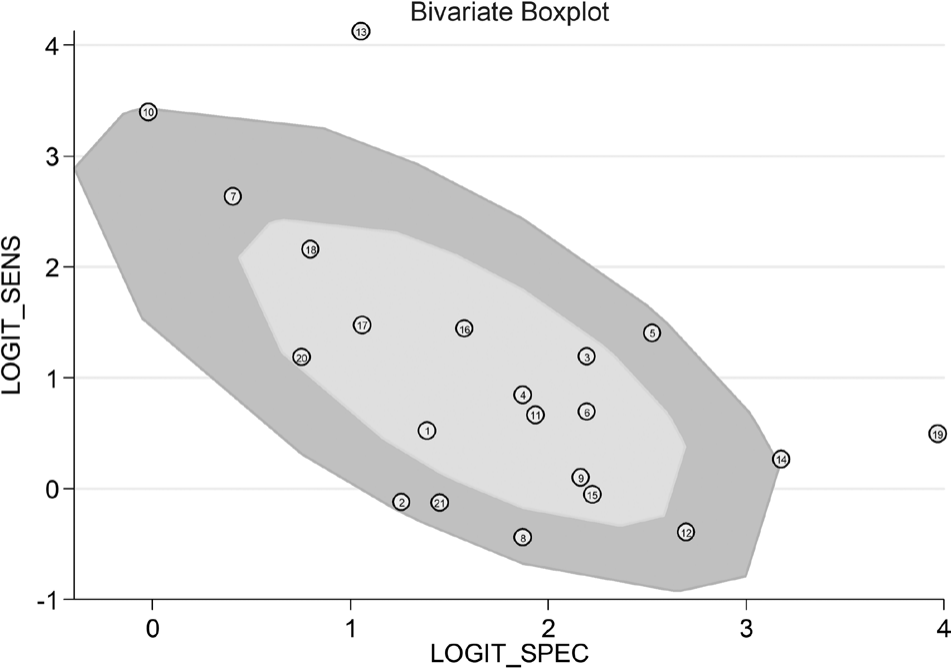
Heterogeneity test of all included studies by bivariate boxplot.

The Cochran Q (chi-square) and *I*^2^ tests were used to evaluate heterogeneity between the included studies. Among these studies, the Cochran *Q* = 284.925 (*P* ≤ .001), *I*^2^ = 99 (95% CI = [99–100]), indicating the existence of significant heterogeneity. Furthermore, a graphic method by bivariate boxplot was used to estimate the heterogeneity. As shown in Figure 3, 3 out of 21 studies fell outside the boxplot, consistently indicated that there may be heterogeneity among included studies.

### 3.5. Diagnostic performance

**Figure 4.**
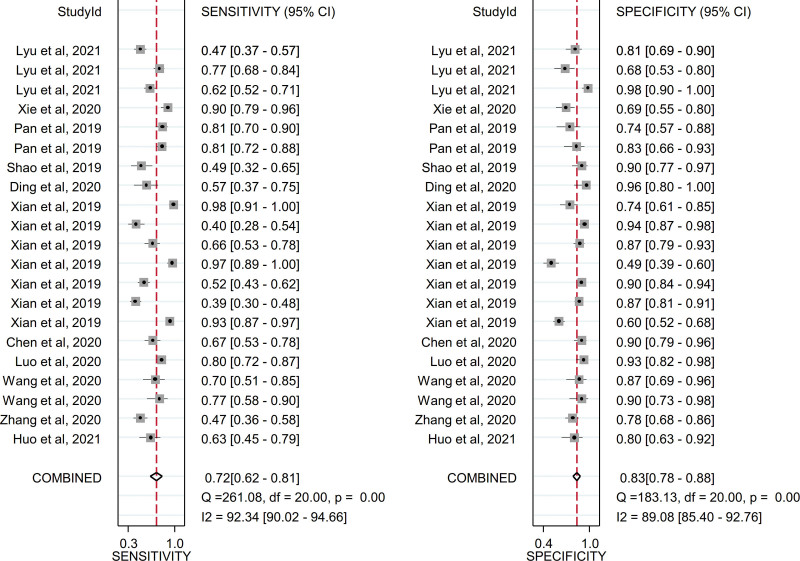
Forest plot of sensitivity and specificity for the diagnosis of circulating exosomal circRNA in malignancies.

**Figure 5.**
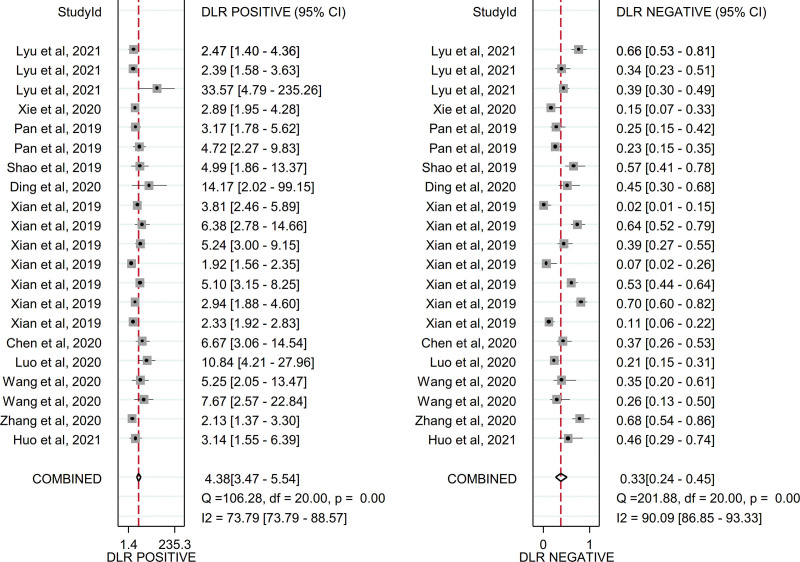
Forest plot of DLR+ and DLR− for the diagnosis of circulating exosomal circRNA in malignancies. DLR = diagnostic likelihood ratio.

**Figure 6.**
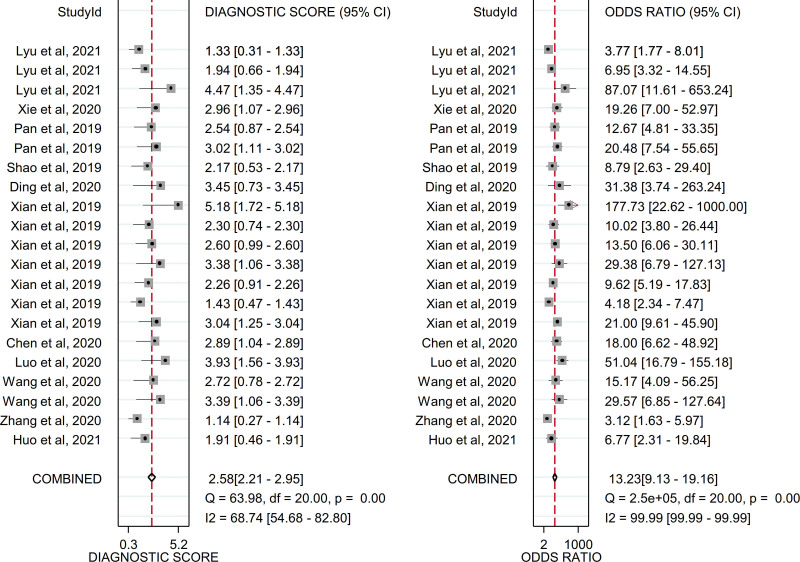
Forest plot of diagnostic score and diagnostic odds ratio of circulating exosomal circRNA in malignancies.

**Figure 7.**
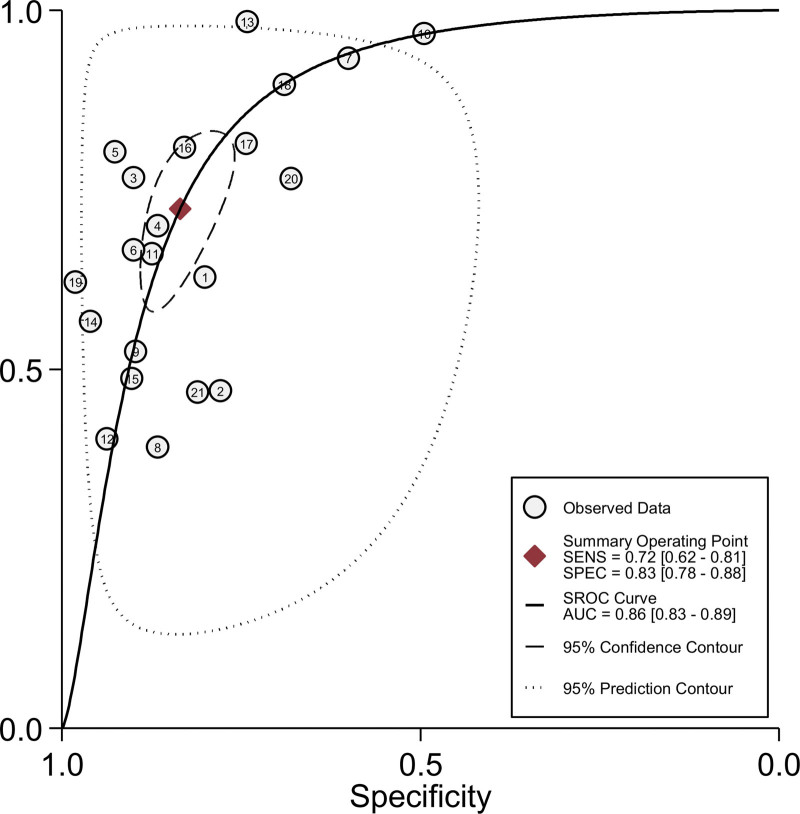
The SROC curve and pooled AUC value for the diagnosis of circulating exosomal circRNA in malignancies. AUC = area under the curve, SROC = summary receiver operator characteristic.

Due to the existence of significant heterogeneity, a random effects model was adopted to estimate the diagnostic effect values of circulating exosomal circRNAs for malignancies. The pooled sensitivity and specificity were 0.72 (95% CI, 0.62–0.81) and 0.83 (95% CI, 0.78–0.88), respectively and the forest plot were shown in Figure 4. The pooled DLR+ and DLR− were 4.38 (95% CI, 3.47–5.54) and 0.33 (95% CI, 0.24–0.45), respectively and the forest plot were shown in Figure 5. The pooled diagnostic score and diagnostic odds ratio were 2.58 (95% CI, 2.21–2.95) and 13.23 (95% CI, 9.13–19.16), respectively and the forest plot were shown in Figure 6. There was significant heterogeneity between included studies in terms of sensitivity (*Q* = 261.08, *P* ≤ .001, *I*^2^ = 92.34), specificity (*Q* = 183.13, *P* ≤ .001, *I*^2^ = 89.08), DLR+ (*Q* = 106.28, *P* ≤ .001, *I*^2^ = 73.79), DLR− (*Q* = 201.88, *P* ≤ .001, *I*^2^ = 90.09), diagnostic score (*Q* = 63.98, *P* ≤ .001, *I*^2^ = 68.74) and diagnostic odds ratio (2.5^5^, *P* ≤ .001, *I*^2^ = 99.99).In addition, SROC curve was constructed and as shown in Figure 7 the pooled AUC was 0.86 (95% CI, 0.83–0.89). These results of pooled analysis suggested a favorable diagnostic efficacy of circulating exosomal circRNAs for malignancies.

### 3.6. Meta-regression analysis and subgroup analysis

In order to further explore the source of heterogeneity, a meta-regression analysis was performed of cancer type, sample size, sample type, study quality and data source. Disappointingly, the results of meta-regression analysis showed that the p values of the subgroup analyses were greater than 0.10, suggesting an insignificant impact of cancer type (*P* = .7564), sample size (*P* = .6105), sample type (*P* = .9545), study quality (*P* = .6105), and data sources (*P* = .7829) on the pooled results.

The diagnostic effect values of subgroup analyses stratified by cancer type, sample size, sample type, study quality and data sources were shown in Table 4. There was no observable difference in the diagnostic performance of circulating exosomal circRNAs between lung cancer and digestive system tumors in terms of sensitivity (0.74 [95% CI, 0.55–0.87] vs. 0.71 [95% CI, 0.59–0.80]), specificity (0.82 [95% CI, 0.74–0.89] vs. 0. 84 [95% CI, 0.75–0.90]), DLR+ (4.20 [95% CI, 3.14–5.61] vs. 4.34 [95% CI, 2.89–6.50]), DLR− (0.32 [95% CI, 0.18–0.56] vs. 0.35 [95% CI, 0.25–0.48]), DOR (13.11 [95% CI, 7.40–23.23] vs. 12.50 [95% CI, 7.46–20.95], and AUC (0.86 [95% CI, 0.83–0.89] vs. 0.85 [95% CI, 0.81–0.88]). However, the range of 95% CIs of theses diagnostic effect values in the digestive system tumor subgroup were narrower than those in the lung cancer subgroup, indicating higher stability of diagnostic performance of circulating exosomal circRNAs for digestive system tumors. Similarly, the results of other subgroup analyses based on sample size, sample type, study quality and data source illustrated that there was no significant difference in the diagnostic power between the subgroups.

**Table 4:** The results of subgroup analysis and meta-regression analysis.

Subgroup	Number of studies	Sensitivity (95% CI)	Specificity (95% CI)	DLR+ (95% CI)	DLR− (95% CI)	DOR (95% CI)	AUC (95% CI)	*P* value
Types of cancer								
Osteosarcoma	1	0.6283	0.7996	3.1358	0.4648	6.7461	0.7780	.7564
Multiple myeloma	1	0.8073	0.9176	9.7997	0.2100	46.6653	0.9240	
Lung cancer	11	0.74 (0.55–0.87)	0.82 (0.74–0.89)	4.20 (3.14–5.61)	0.32 (0.18–0.56)	13.11 (7.40–23.23)	0.86 (0.83–0.89)	
Digestive system tumor	8	0.71 (0.59–0.80)	0.84 (0.75–0.90)	4.34 (2.89–6.50)	0.35 (0.25–0.48)	12.50 (7.46–20.95)	0.85 (0.81–0.88)	
Sample size								
<100	5	0.63 (0.53–0.71)	0.88 (0.82–0.92)	5.31 (3.41–8.26)	0.42 (0.33–0.54)	12.52 (6.97–22.51)	0.88 (0.85–0.90)	.6105
>100	16	0.75 (0.62–0.85)	0.82 (0.75–0.87)	4.15 (3.20–5.38)	0.30 (0.20–0.46)	13.62 (8.68–21.37)	0.86 (0.83–0.89)	
Sample type								
Plasma	7	0.64 (0.55–0.72)	0.88 (0.80–0.93)	5.29 (3.09–9.06)	0.41 (0.32–0.52)	12.90 (6.63–25.09)	0.82 (0.78–0.85)	.9545
Serum	14	0.76 (0.62–0.86)	0.81 (0.74–0.87)	4.06 (3.17–5.19)	0.29 (0.18–0.46)	13.91 (8.67–22.32)	0.86 (0.82–0.89)	
Study quality								
Low	5	0.63 (0.53–0.71)	0.88 (0.82–0.92)	5.31 (3.41–8.26)	0.42 (0.33–0.54)	12.52 (6.97–22.51)	0.88 (0.85–0.90)	.6105
High	16	0.75 (0.62–0.85)	0.82 (0.75–0.87)	4.15 (3.20–5.38)	0.30 (0.20–0.46)	13.62 (8.68–21.37)	0.86 (0.83–0.89)	
Data source								
Collection from article	8	0.70 (0.58–0.80)	0.85 (0.74–0.91)	4.52 (2.83–7.22)	0.36 (0.26–0.50)	12.70 (7.20–22.41)	0.85 (0.81–0.88)	.7829
Extraction from ROC	13	0.74 (0.59–0.85)	0.83 (0.76–0.88)	4.38 (3.33–5.75)	0.31 (0.19–0.50)	14.09 (8.41–23.63)	0.86 (0.83–0.89)	

*P* value is the *P* value of meta-regression analysis.

AUC = area under curve, CI = confidence interval, DLR− = negative diagnostic likelihood ratio, DLR+ = positive diagnostic likelihood ratio, DOR = diagnostic odds ratios.

### 3.7. Publication bias

**Figure 8.**
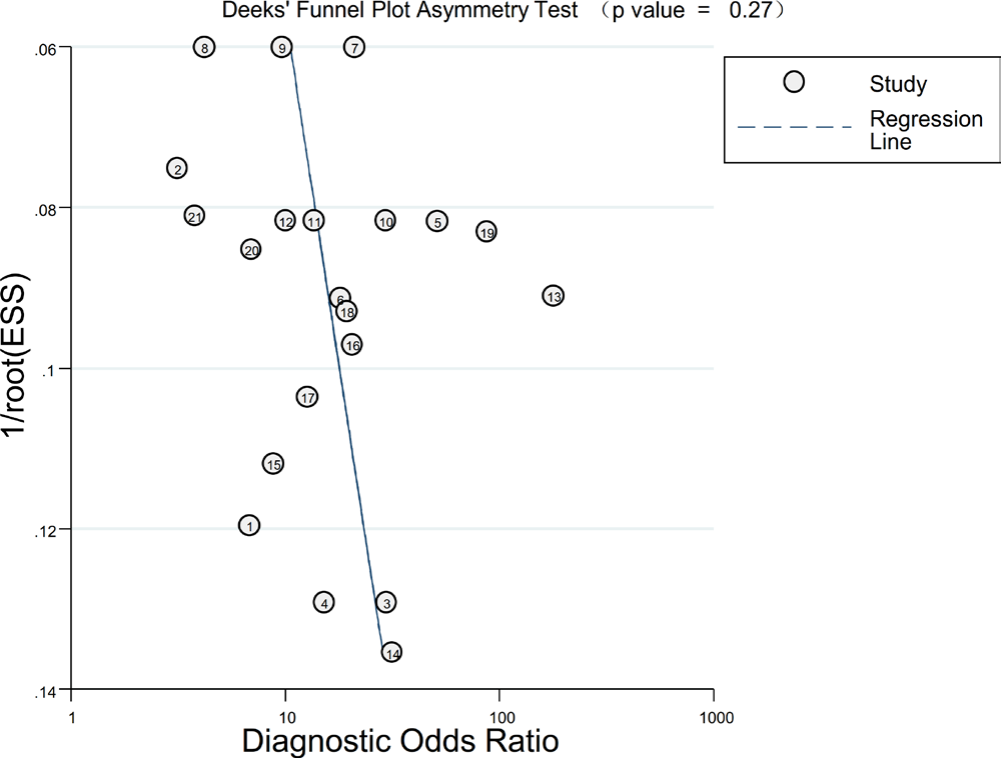
The assessment of publication bias among the included studies by Deek’s funnel plot asymmetry test.

Publication bias of included studies was assessed by the Deek’s funnel plot asymmetry test, and as shown in Figure 8, the *P* value of was .27, which is greater than .05, indicating the absence of potential publication bias.

### 3.8. Clinical significance of the study

**Figure 9.**
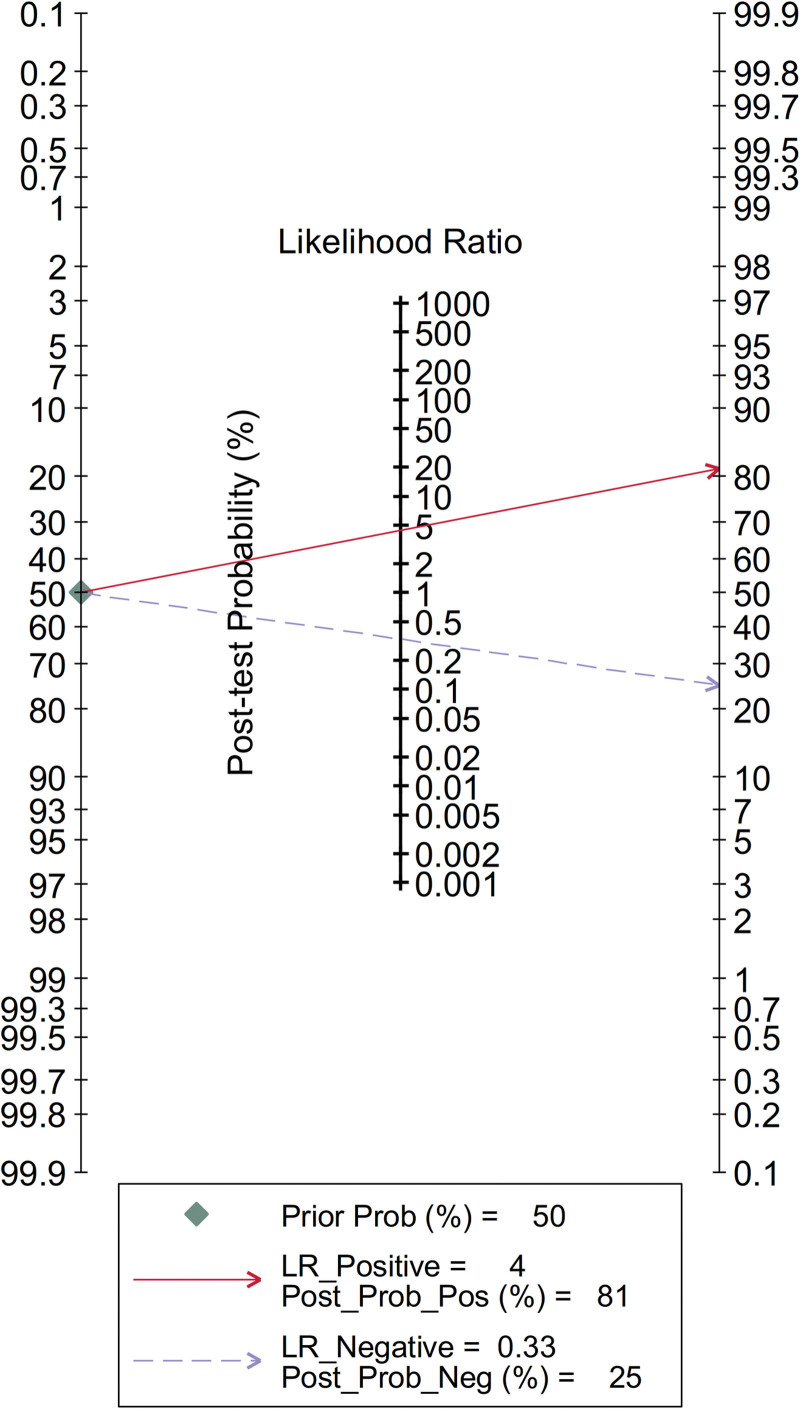
The estimating of the clinical significance in malignance diagnosis of circulating exosomal circRNA by Fagan plot.

In clinic, physicians expect to estimate the probability of morbidity for suspicious patients according to the detection of abnormal expression of circulating exosomal circRNAs combined with the patient’s history and physical signs. To explore the clinical significance of this study, the Fagan plot was constructed to explain the interaction between pretest probability, likelihood ratio and post-test probability. As shown in Figure 9, assuming that the pretest probability was 50%, the post-test probability was 81% based on the positive diagnostic likelihood ratio of 4, indicating a promising clinical diagnosis value of circulating exosomal circRNAs for malignancies.

**Figure 10.**
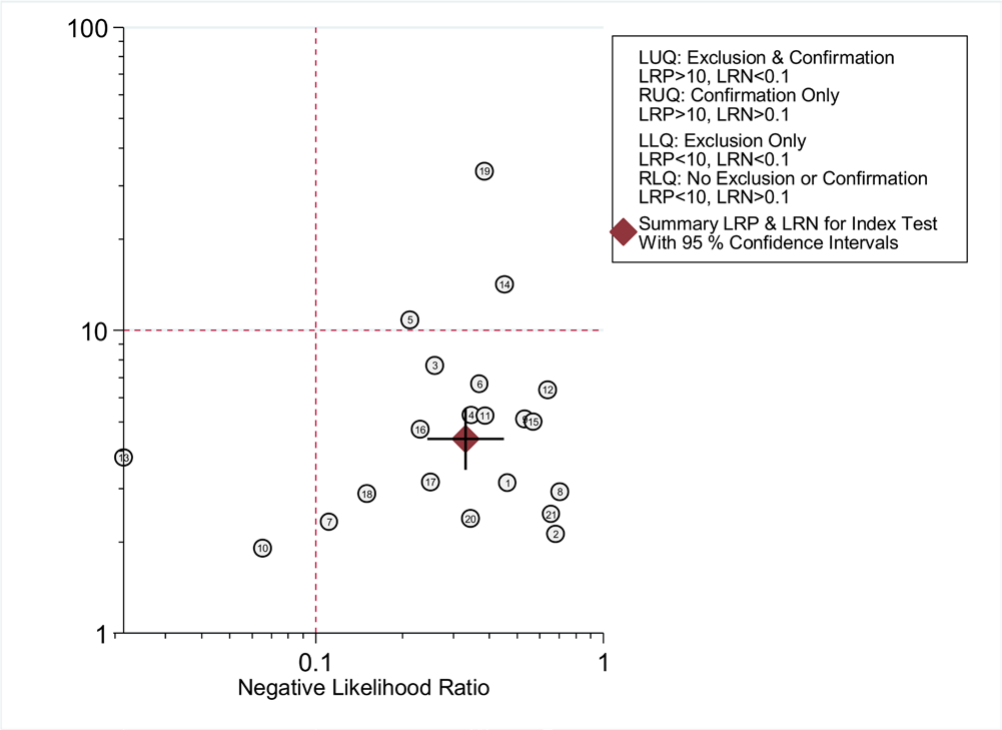
The likelihood ratio scattergram to estimate the clinical significance in malignance diagnosis of circulating exosomal circRNA.

Furthermore, the likelihood ratio scattergram was performed. The graph is divided into 4 quadrants, that is, upper left limit (LUQ), upper right limit (RUQ), lower left limit (LLQ) and lower right limit (RLQ). In LUQ, DLR+ was greater than 10 and DLR− was less than 0.1, indicating that malignancies can be confirmed and excluded by the test. In RUQ, DLR+ was greater than 10 and the DLR− was greater than 0.1, indicating that malignancies can only be confirmed. In LLQ, DLR+ was less than 10 and DLR− was less than 0.1, indicating that malignancies can only be excluded. In RLQ, DLR+ was less than 10 and DLR− was greater than 0.1, indicating that malignancies can neither be confirmed nor be excluded. As shown in Figure 10, the diagnostic power of circulating exosomal circRNAs was limited in clinical confirmation and exclusion of malignancies.

## 4. Discussion

CircRNAs, characterized with covalently closed loop and great stability, function as post-transcriptional regulators, contributing to tumor angiogenesis and metastasis. Aberrant expression of circRNAs was often detected in plasma and serum of patients with cancer, indicating the potential diagnostic value of circRNAs in malignancies. CircRNAs have been found to be enriched, stable and ubiquitous in serum and plasma exosomes,^[12]^ which have good secretory characteristics and stable vesicular structure. Thus, the detection of a specific circRNA in circulating exosomes of cancer patients provides new ideas and research methods for application as a promising noninvasive diagnostic biomarker for cancers.^[4]^

In the recent years, studies emerged to assess the diagnostic performance of circulating exosomal circRNAs in malignancies. One published article explore the role of circ_0056285 in osteosarcoma, in which dual-luciferase reporter assay, RNA immunoprecipitation assay and RNA pull-down assay were conducted to illustrate the interaction among circ_0056285, miR-1244 and tripartite motif containing 44. The diagnostic value of serum exosomal circ_0056285 was evaluated by ROC curve with a AUC of 0.778.^[34]^ Similarly, circulating exosomal circMYC was a promising biomarker with a high AUC value of 0.924 for multiple myeloma patients.^[36]^ The serum exosomal circSATB2 exerted a moderate diagnostic powder for lung cancer based on a moderate AUC value of 0.660, while it presented higher diagnostic value for distinguishing metastatic lung cancer patients from non-metastatic lung cancer based on a AUC of 0.797.^[18]^ In addition, there were studies reporting varied diagnostic performance of specific circulating exosomal circRNAs for gastric cancer, colorectal cancer and hepatocellular carcinoma.

In our study, a systematic literature review and meta-analysis was conducted for the first time to provide a comprehensive overview on the diagnosis accuracy of circulating exosomal circRNAs in malignancies. A number of 11 articles comprising 21 studies were included with a total of 1609 cases involving 6 types of malignant tumors and 1498 controls. Fourteen kinds of serum or plasma exosomal circRNAs were detected and evaluated, including circ_0056285, circSATB2, circ_0014235, circ_0025580, circMYC, circ_0051443, circ_0047921, circ_0056285, circ_0007761, circMEMO1, circ_0065149, circ-0004771, circ_0101802 and circ_0070396. In all included studies, serum or plasma exosomes were isolated with the authoritative methods, and isolated exosomes were identified by TEM and western blot assay of exosome marker in 8 out of 11 articles. The expression level of exosomal circRNAs was detected by quantitative real-time polymerase chain reaction (qRT-PCR). Thus, the results of included studies were credible and practicable. The pooled sensitivity and specificity were 0.72 (95% CI, 0.62–0.81) and 0.83 (95% CI, 0.78–0.88), respectively. The pooled diagnostic odds ratio was 13.23 (95% CI, 9.13–19.16). In addition, SROC curve was constructed and the pooled AUC was 0.86 (95% CI, 0.83–0.89). These results of the pooled analysis suggested a favorable diagnostic efficacy of circulating exosomal circRNAs for malignancies.

In the recent years, several systematic review were published to comprehensively evaluate the diagnostic accuracy of circRNA expression profiles for cancer. In 2019, Tan et al^[42]^ assessed the overall diagnostic efficiency of circRNAs in tissues and plasma, reporting that the pooled sensitivity, specificity and area under the curve were 0.79 (95% CI: 0.73–0.84), 0.73 (95% CI: 0.67–0.79) and 0.83 (95% CI: 0.79–0.86), respectively. In the same year, Zhao et al^[43]^ revealed the diagnostic value of circular RNAs in colorectal cancer in a systematic review and meta-analysis. The pooled sensitivity, specificity, and AUC were 0.78 (95% CI: 0.70–0.84), 0.71 (95% CI: 0.65–0.76) and 0.80 (95% CI: 0.76–0.83), respectively. In 2018, Wang et al^[5]^ collected and examined all the evidence on the potential role of circRNA as novel biomarker in human cancers. False-negative results are a significant concern in clinical practice and can have adverse consequences for patients. Several factors can contribute to false-negative results in serum exosomal circRNA analysis, including sample quality, sample size, disease stage, and genetic heterogeneity. Additionally, technical issues such as poor RNA extraction, inadequate normalization, and sequencing errors can also lead to false-negative results. For the first time, we provided an updated overview on the diagnosis accuracy of circulating exosomal circRNA for malignancies, and the results revealed that circulating exosomal circRNA had better diagnostic performance than serum and tissue circRNA expression profiles. Our study provided the evidence supporting the potential clinical diagnosis value of circulating exosomal circRNA as a simple and convenient method of liquid biopsy.

During the systematic review and meta-analysis on diagnostic performance, many eligible original researches only provide ROC curves, not accessible to enough primary data that are required for analysis. The exclusion of these “not enough” studies may bring selection bias into the pooled analysis and discount the reliability of the pooled results. Engauge Digitizer is a credible digitizing software, which was commonly used for reading curves and extracting raw data^[24]^ and in our previous study we used Engauge Digitizer to read the Kaplan–Meier curves to obtain information of survival.^[25]^ Youden’s index has been developed to determine the optimal sensitivity and specificity values for quantitative diagnostic tests.^[26]^ In this meta-analysis, 11 eligible articles relevant to the topic were enrolled, however, only 5 articles provided the values of Sen and Spe,^[37–41]^ while only ROC curves and AUC values were available for the other 6 articles.^[18,22,23,34–36]^ We obtained the optimal Spe and Sen of the former 5 articles through reading ROC curves by Engauge Digitizer combined with calculating the maximum of Youden’s index, which produced results essentially in agreement with the corresponding values available in the articles. Therefore, we considered the method of data extraction credible and convenient, and in this way we obtained the optimal Sen and Spe for the other 6 articles. A comprehensive analysis of a total of 11 original articles was performed and we further conducted the meta-regression and subgroup analysis by data source. The results showed that data extraction in this way did not bring heterogeneity into the meta-analysis.

Notwithstanding, the main limitations of the meta-analysis should be acknowledged. Firstly, although the data extraction through digitizing software-Engauge Digitizer combined with the maximum of Youden’s index was convenient and credible, it is undeniably limited to a certain extent. Secondly, significant heterogeneity was detected among included studies. In order to further explore the source of heterogeneity, the meta-regression analysis and subgroup analyses were performed by cancer type, sample size, sample type, study quality and data source. Disappointingly, the source of heterogeneity could not be identified from these confounding factors.

## 5. Conclusions

In conclusion, our study evaluated the diagnostic power of circulating exosomal circRNAs in 6 types of cancer by synthesis of published data comprising 21 studies from eleven articles, supporting circulating exosomal circRNAs as a promising noninvasive diagnostic biomarkers for malignancies. Further evidence are required to investigate the diagnostic accuracy of a specific circulating circRNA in one specific type of cancer stratified by age, gender and tumor stage, thus paving the way for clinical diagnostic application of circulating exosomal circRNA. Meanwhile, molecular biology experiments are needed to clarify the mechanism underlying the interaction among circRNA, miRNA and mRNA.

## Acknowledgments

We would like to thank Jianbo Tan from Changsha University of Science & Technology for his valuable lessons in the usage of digitizing software-Engauge Digitizer.

## Author contributions

**Conceptualization:** Xia Yuan.

Data curation: Xia Yuan.

Formal analysis: Xia Yuan.

Funding acquisition: Xia Yuan.

Investigation: Ye Mao.

Methodology: Ye Mao, Shuangyan Ou.

Project administration: Ye Mao.

Resources: Ye Mao.

Software: Ye Mao.

Validation: Shuangyan Ou.

Visualization: Shuangyan Ou.
